# Annotations

**Published:** 1887-05-28

**Authors:** 


### ANNOTATIONS.

**The Hospitals Association.**

THE Hospitals Association has just brought its session to a close, a session which has been in every way significant of progress and of hope. The Association has entirely surmounted its early difficulties, and is fully established in the confidence of experienced and trusted hospital managers. The supporters and administrators of medical charities, like other persons, see the inestimable advantages of frequent conference one with another, and with thoughtful outsiders. Few men, even among hospital officials, are possessed of all wisdom and all knowledge. The sober and the experienced among these are like Newton and other famous students, the more they know, the more they see the vast regions of darkness and uncertainty which lie round about them; and hence they love to take frequent counsel with their follows. Self-satisfied and ignorant persons there may be who hold sulkily aloof, but these are few, and are constantly diminishing in numbers and in importance. The Association has discussed several important subjects during the winter and spring, with the result of widening and deepening general knowledge. It has developed its organisation, with the view of entering upon new and enlarged fields of labour. It aims and resolves to extend its influence not only to all the hospitals of every class in Great Britain, but also to all persons who may need or desire to be in any way connected with the hospital system. If it continues on the lines laid down by its distinguished President, and endorsed by its representatives at the annual meeting, there can be no doubt that it has before it a future of great and growing usefulness.

**The Hospitals Weeks.**

The hospitals are rousing themselves to make known their needs and claims to the public with right good will. With few exceptions the seventy medical charities of the metropolis are combining for an organised and vigorous campaign. There can only be one result where the invaders are determined to conquer, and the invaded are anxious to surrender. There is not the least doubt that all ranks and conditions of Englishmen are in the fullest possible agreement with hospitals and their noble work. If there be lack of adequate help, the lack arises neither from want of sympathy nor from want of means, but simply from want of knowledge and clear understanding. The hospitals are now organising themselves into a grand union, and are taking such steps to enlighten the public mind, that results of the most important and far-reaching character may be confidently expected. They are enlisting and sending forth an army of speakers, whose names alone, like Joshua's trumpets, will make the walls of the Jericho of Wealth to fall down. The Archbishop of Canterbury, as is in every way fitting, is placed at the head of the list. Cardinal Manning, Dr. Allon, and Mr. Spurgeon are also among the speakers, and give assurance that in deeds of charity men forget they were enemies before. The Prime Minister gives the weight of his great name and position, and is well flanked by distinguished members of both Houses of Parliament. Sir Andrew Clark and other eminent representatives of the medical profession lend the authority of scientific medicine to the movement] Nothing is wanting to the imposing character of the demonstration, and we are sanguine that as are the generous and enthusiastic efforts that will be made, so will be the large and liberal response of a cordial and approving people. We shall publish an extra double number next week, in support of the Hospital Sunday and Saturday Funds, as announced in our advertising columns.

**Do Nothings.**

Sir Edmund Currie, at the meeting of the Council of the Hospital Sunday Fund on Friday, reported that the University College Hospital, King's College Hospital, Charing Cross Hospital, and Westminster Hospital not only declined to help to awaken increased interest in the work of all the hospitals of London, but that some or all of them were quietly opposing the efforts of the Council to increase the contributions on Hospital Sunday. Such a state of affairs is nothing less than a grave scandal, affecting the interests of the public, and one which the philanthropic portion of it, we believe, will not be slow to notice and resent. There is, of course, an abundance of half-hearted people in the world, but when four out of seventy metropolitan hospitals set themselves up, not only against their own interests, but against those of the remainder, prompt action should be taken to mark with disapprobation so selfish and destructive a policy. When the authorities of great hospitals like the London, St. Mary's, St. George's, the Seamen's, the German, and indeed all the other leading medical institutions, are exerting themselves to arouse public interest, and so to benefit each institution by increasing the amount of the Hospital Sunday collections, it is regretable indeed to find that four general hospitals—University College, King's College, Charing Cross, and Westminster—should take a contrary course, or by inaction do their best to prevent the collection of an adequate sum on Hospital Sunday. We would therefore publicly ask the managers of these hospitals for an explanation of their proceedings in this matter, and would point out to them that, as their institutions are situated in relatively wealthy districts of the metropolis, their action has the appearance of being as mistaken as it is selfish, and as selfish as it is unworthy. It behoves them to explain their position without a moment's delay, or we shall not be surprised to find that they will suffer a serious diminution of income and support, until they exhibit more public spirit and generosity towards the inhabitants of the poorer districts of London.

